# Supplementary material for: Predictors of low birthweight and comparisons of newborn birthweights among different groups of maternal factors at Rev. John Chilembwe Hospital in Phalombe district, Malawi: A retrospective record review
Source: PLoS One. 2024 Aug 29;19(8):e0291585. doi: 10.1371/journal.pone.0291585 (PMC11361679; doi:10.1371/journal.pone.0291585)
Supplement: Attachment — Submitted filename: Responses to Reviewers.docx [file pone.0291585.s002.docx]

RESPONSES TO REVIEWERS’ COMMENTS

| **Page #** | **Comments by Reviewer 1** | **Authors’ Response** |
| --- | --- | --- |
|  | The manuscript entitled “Comparisons of newborn birthweights with maternal factors at Phalombe District Hospital, Malawi: a retrospective record review” compares the birthweights of infants born to different maternal groups in Malawi, Africa. Using data from the birth registry of Phalombe District Hospital, the authors examine the impact of four maternal variables, age, HIV status, parity, and obstetric complications, on birthweight. This study holds significance as it highlights the factors that can have an impact on birthweight in Malawi. Some recommendations to enhance the manuscript are outlined below: | Thank you.  This title has now been revised to “Predictors of low birthweight and comparisons of newborn birthweights with maternal factors at Phalombe District Hospital, Malawi: a retrospective record review.” (See Lines 1 to 3) |
|  | The background is focused on low birth weight (LBW) in infants and maternal risk factors for LBW; however, comparisons have been made on median birthweight in different maternal groups. It would be good to check if the prevalence of LBW was different among the maternal categories presented. Also, rather than focusing solely on LBW, explore these differences using data on infants classified as small-for-gestational-age: SGA (birthweight below the 10th percentile for gestational age). For instance, a newborn with a birthweight of 2,400 grams could fall into the SGA, AGA, or LGA categories, and the associated outcomes would vary depending on the specific category the newborn belongs to. Research has shown that maternal predictors also can vary based on birthweight categorisations. I think additional analyses that integrate data on birthweight standardised for gestational age would enhance the interpretability of the findings. | Thank you.  We have done the comparisons about the prevalence of LBW among different maternal categories (See Table 1, Lines 219 to 220).  This study dwelt on LBW as such we have included 'lack of classification of SGA' as a limitation and calling on future research to consider addressing this gap (See Lines 548 to 549). |
|  | The authors have used non-parametric tests as the independent variables did not follow a normal distribution; however, there is no mention of whether the outcome variable (birthweight) was normally distributed. Did the authors consider using multivariable logistic regression analysis, which can be used to assess the relationship between several independent variables and outcome variables such as LBW or SGA? It also allows the assessment of independent relationships while adjusting for potential confounders. Logistic regression assumes that the log odds (logit) of the dependent variable is a linear combination of the independent variables. This means that the relationship between the independent variables and the log odds of the outcome variable is linear, but it does not require the independent variables themselves to be normally distributed. | Thank you.  We have included a statement to show that the outcome variable was skewed. See Table 3, Lines 249 to 250.  We have performed logistic regression analyses (See Table 4, Lines 329 to 330). |
|  | Only mean ranks of birthweight are presented in the results. Suggest providing the birthweights representing those mean ranks for meaningful results. | Thank you.  We have replaced mean ranks with median birthweights which are more meaningful. We also changed the method of data analysis. We have now used Median Test as the data contains some outliers. |
|  | Are there any other studies which support the finding that “there were no statistically significant differences in mean ranks of newborn birthweights born to HIV positive and HIV negative women”? I would take caution on this result. Could this have resulted due to confounding factors, importantly gestation length? | Thank you.  It is important to take note that we compared birthweights with maternal factors using one-way Median test. Each test was independent of the other variables as such these comparisons did not control for any confounding factors. We have, however, taken note of this input and we have included gestation length (in weeks) which has been categorized into term (37 – 41.6 weeks), preterm (≤36.6 weeks) and postterm (≥42 weeks) (See Lines 142 to 144). However, in our logistic regression analysis, this has been taken care of where gestation age and other variables were adjusted for. After performing logistic regression analysis still shows that being HIV+ is not statistically associated with LBW deliveries at univariate level (OR = 1.01, 95% CI 0.61 - 1.67, p = .979) (See Table 4, Lines 329 to 330).  **For your information, other studies have found similar results. For example, a study by Habib et al (Habib NA, Daltveit AK,**  [**Bergsjø**](https://obgyn.onlinelibrary.wiley.com/authored-by/Bergsj%C3%B8/P) **P,** [**Shao**](https://obgyn.onlinelibrary.wiley.com/authored-by/Shao/J) **J,** [**Oneko**](https://obgyn.onlinelibrary.wiley.com/authored-by/Oneko/O) **O  &** [**Lie**](https://obgyn.onlinelibrary.wiley.com/authored-by/Lie/RT) **RT. Maternal HIV status and pregnancy outcomes in northeastern Tanzania: a registry-based study. BJOG. (2008) 115 (5), 616 – 624.** <https://doi.org/10.1111/j.1471-0528.2008.01672.x>) found that treated HIV-infected women had a risk similar to that of the HIV-uninfected women for all outcomes (perinatal mortality, prematurity, SGA, birthweight), except for low Apgar score. All HIV+ women in our study were on ART treatment (as indicated in the maternity registers).  On the contrary, a study by Wedi et al. found that maternal HIV infection in women who have not received antiretroviral therapy is associated with preterm birth, low birthweight, small for gestational age, and stillbirth, especially in sub-Saharan Africa. No association was identified between maternal HIV infection and very preterm birth, very small for gestational age, very low birthweight, miscarriage, or neonatal death, although few data were available for these outcomes. Correction for confounders did not affect the significance of these findings. (Wedi COO, Kirtley S, Hopewell, S, Corrigan, R, Kennedy SH, Hemelaar J. Perinatal outcomes associated with maternal HIV infection: a systematic review and meta-analysis. (2016) 3 (1), E33-E48. The Lancet HIV. [<https://doi.org/10.1016/S2352-3018(15)00207-6>)](https://doi.org/10.1016/S2352-3018(15)00207-6)). Wedi et al |
|  | Did the authors have data on other important maternal risk factors for LBW and SGA, such as smoking, drug abuse, being underweight, etc. There should be at least a discussion on those factors. | Thank you.  Data on smoking, drug abuse and being underweight, etc are not captured in the maternity registers in Malawi as such we did not have such data at our disposal. We have, however, included this as study limitation (See Lines 530 to 532). |
| **Page #** | **Comments by Reviewer 2** | **Authors’ Response** |
|  | Thank you for the opportunity to review this paper. The aim was to compared newborn birthweights with maternal risk factors at a hospital in Malawi. It was a retrospective study of 1308 women who gave birth over a 6 month period. | Thank you. |
|  | How accurate was the recording of birthweight and the different maternal conditions especially pre eclampsia, sepsis and APH. There are no definitions provided or any sense of magnitude. Would one episode of APH at 24 weeks classify as APH in a woman who have birth at term? What magnitude of sepsis was used, what definition and when did the sepsis occur (I presume during pregnancy but this is not clear). Why is (pre) eclampsia written with the brackets? I have never seen this before. | Thank you.  The recording of birthweight was accurate as this was done soon after delivery before the baby was taken to postnatal ward. In additional, all the weights were recorded in grams and those newborns who had missing birth weight data were excluded from the analysis. For the maternal conditions (pre-eclampsia, sepsis and APH), the register that we extracted data from already had these conditions listed and any woman who presented with such a condition during labour and delivery, was recorded accordingly. For the definitions of the maternal conditions, this has been noted and operational definitions have been provided (See Lines 128 to 144).  (Pre) Eclampsia has been written with brackets because this is how the register from where the data was extracted has been designed. This means that any woman with Pre-eclampsia or with Eclampsia is recorded in the same category.  We have included the magnitude of this problem in the introduction (See Lines 69 to 71). You will also find the findings on the same in results section. We defined obstetric complications as pregnant women having (pre)-eclampsia, antepartum hemorrhage and sepsis. These were selected due to their effect on LBW from previous studies. We did not want to include complications that have no scientific backing as factors associated with LBW.  As you will have noted from the study, we collected data retrospectively from maternity registers. This made it impossible for us to know when the APH episodes started occurring during pregnancy as the registers do not specify this. One thing for sure is that episodes occurring during birth time were recorded. |
|  | These conditions were then coded dichotomously – absent or present. This feels very blunt. How can we be sure that this is sensitive enough? | Thank you.  We have revised the way we coded obstetric complications. This has now been categorized and coded as below: women with pre-eclampsia, including those with eclampsia (1), women with antepartum hemorrhage, including those with sepsis (2) and control group [refers to women with no pre-eclampsia/eclampsia, antepartum hemorrhage and sepsis] (3). See Lines 139 – 141.  NOTE: 1 woman had sepsis and delivered a LBW baby. This has been combined with APH. Secondly, the maternal registers just have column labelled '(pre)-eclampsia.' This is a column that captures women with pre-eclampsia and eclampsia. Health workers do not specify if what they are recording is either pre-eclampsia or eclampsia. That is why we have coded it the way we have. |
|  | Was the use of ART considered in the HIV positive women and again, the range of health care status in women with HIV. Just being positive feels a bit blunt. | Thank you.  All HIV+ women in our study were on ART. We have added a statement to indicate that (See Lines 471 to 474). |
|  | Was the mode of birth considered? What is the rate of induction of labour or elective caesarean section in this hospital? | Thank you.  We have included mode of delivery and it has been categorized as normal deliveries, caesarean section deliveries and assisted vaginal deliveries (See Lines 131 to 135). |
|  | How many women/babies had missing data? | Thank you.  We have included the statement below under the results section.  Eighty-seven (87) women did not meet the inclusion criteria as 69 had their gestation weeks either unrecorded or unclear, 13 had their parity unclear, 4 had their ages unrecorded and 1 had her HIV status unrecorded. One hundred eighty-eight (188) newborns were not included as 47 had birthweights unrecorded, 104 were twins and 37 were stillbirths (macerated stillbirths = 14 and fresh stillbirths = 23) (See Lines 200 to 204). |
|  | I was surprised that stillbirths were excluded. Surely stillbirth is an example of an impact of low birth weight? | Thank you.  We excluded stillbirths to be in line with the definition of incidence of LBW. UNICEF and WHO define incidence of LBW in a population as the percentage of **live births** that weigh less than 2,500g out of the total of **live births** during the same time period (United Nations Children’s Fund and World Health Organization. Low birthweight: Country, regional and global estimates. New York: UNICEF; 2004) (See Lines 112 to 114). |
|  | How was gestation controlled for in the study? I cannot see any information about gestation or how this was considered in the analysis. This seems to be a major flaw and an explanation that is not discussed. | Thank you.  Please note that gestation has been controlled for in our study when conducting multivariate regression analysis (See Table 4, Lines 329 to 330). |
