# Supplementary material for: Predictors of low birthweight and comparisons of newborn birthweights among different groups of maternal factors at Rev. John Chilembwe Hospital in Phalombe district, Malawi: A retrospective record review
Source: PLoS One. 2024 Aug 29;19(8):e0291585. doi: 10.1371/journal.pone.0291585 (PMC11361679; doi:10.1371/journal.pone.0291585)
Supplement: Attachment — Submitted filename: Responses to Reviewers.docx [file pone.0291585.s003.docx]

RESPONSES TO REVIEWERS’ COMMENTS

| **Page #** | **Comments by Reviewer 1** | **Authors’ Response** |
| --- | --- | --- |
|  | Thank you for the revisions on the manuscript. I appreciate the effort you put into addressing my feedback. I have no further comments. | Thank you. |
| **Page #** | **Comments by Reviewer 3** | **Authors’ Response** |
|  | Thank you for the opportunity to review the manuscript. The manuscript aims to identify the predictors of low birth weight based on retrospective data from birth registers at Phalombe district hospital. The research question and methodology are clear, and the discussion is detailed. However, I have a few questions and minor recommendations. | Thank you. |
|  | Is Table 2 necessary? Since it is a single row and explained in the text, the table seems redundant. | Thank you. Table 2 has been removed while the text explaining it has been retained (See Page 13, Lines 220 – 222 in the track change version). |
|  | In the introduction (lines 69-71) it is stated that there were 12% LBW deliveries from 10th October to 31st December 2022 and 9% LBW deliveries from 01st January to 31st March. Is there a reason the final sample in this study has a much higher proportion of LBW? | Thank you. We observed a higher prevalence of LBW for the study period of October, 2022 to March, 2023 than 12% and 9% for October to December, 2022 and January to March, 2023, respectively, as reported by the hospital. This difference came about due to data cleaning challenges among midwives. In few instances, midwives had failed to indicate that babies were LBW even though they had entered the newborn birthweights as less than 2,500g. This led to incorrect LBW page summary calculations in the maternity registers, which resulted in lower proportion of LBW. With reference to WHO definition of LBW, we rectified such errors by correctly categorizing LBW as those newborns weighing less than 2,500g since the data on birthweight was available to us in the maternity registers. Poor data quality on LBW was also noted in the Malawi Multiple Indicator Cluster Survey 2019-2020 report, and it recommended cautious use of this data as it was considered an under estimation of the true prevalence (National Statistical Office, 2021) (See Page 20, Lines 352 – 360 in the track change version). |
|  | The authors have clarified the reason for not including the other adverse neonatal outcomes such as stillbirths. However, this is a limitation in the interpretation of some results. Especially in the comparisons between two high risk groups such as 'adolescent girls' and 'women of advanced maternal age' (lines 379-380) or between different obstetric complications, the non-inclusion of other adverse neonatal outcomes limits the inferences. | Thank you. This has been noted but it was necessary for us to stick to the operational definition of incidence of LBW in a population as the percentage of live births that weigh less than 2,500g out of the total of live births during the same period. As a result, we had to exclude the stillbirths. However, we have included this as our study limitation (See Page 26, Lines 499 to 503 in the track change version). |
|  | The discussion and conclusion can be more concise and nuanced. Maternal age is emphasized as an important variable in the discussion without any attempt to explain the why Maternal age is not significant in the multivariate analysis. Maternal age may also be related to other social and economic variables which may affect obstetric complications with complex, intersectional relationships. Hence, more data is needed to identify the interventions in this area. Some recommendations are too generalized and not derived from the study results (lines 431, 581-583). | Thank you. We have revised the conclusion section to make it more concise and nuanced (See Page 27 Lines 506 to 514 in the track change version). We have removed the sub-headings in the discussion section. Generally, its content has been left as it was so that we do not dilute it. We have also explained why maternal age may not have been significant in the multivariable analysis (See Pages 18 and 19, Lines 316 to 334 in the track change version). This statement has been inserted in the discussion section under maternal age. We have removed the recommendations that were deemed too generalized and not derived from the study results. |
